# *Neofusicoccum mediterraneum* Is Involved in a Twig and Branch Dieback of Olive Trees Observed in Salento (Apulia, Italy)

**DOI:** 10.3390/pathogens11010053

**Published:** 2022-01-02

**Authors:** Angela Brunetti, Antonio Matere, Valentina Lumia, Vittorio Pasciuta, Valeria Fusco, Domenico Sansone, Paolo Marangi, Nicola Cristella, Francesco Faggioli, Marco Scortichini, Massimo Pilotti

**Affiliations:** 1 Council for Agricultural Research and Economics (CREA)-Research Centre for Plant Protection and Certification (CREA-DC), Via C.G. Bertero, 22, 00156 Roma, Italy; angela.brunetti@crea.gov.it (A.B.); antonio.matere@crea.gov.it (A.M.); valentina.lumia@crea.gov.it (V.L.); vpasciuta@gmail.com (V.P.); val.fusco94@gmail.com (V.F.); domenico.sansone@crea.gov.it (D.S.); francesco.faggioli@crea.gov.it (F.F.); 2 Terranostra S.r.l.s., Via Monte Grappa, 48, 72021 Francavilla Fontana, Italy; studioterranostra@gmail.com (P.M.); nicolacristella@gmail.com (N.C.); 3 Council for Agricultural Research and Economics (CREA)-Research Centre for Olive, Fruit Trees and Citrus Crops (CREA-OFA), Via di Fioranello, 52, 00134 Roma, Italy; marco.scortichini@crea.gov.it

**Keywords:** olive tree, Botryosphaeriaceae, *Neofusicoccum mediterraneum*, branch and twig dieback, *Xylella fastidiosa*, olive quick decline syndrome OQDS, wilting, canker

## Abstract

Olive trees are infected and damaged by Botryosphaeriaceae fungi in various countries. The botryosphaeriaceous fungus *Neofusicoccum mediterraneum* is highly aggressive and is a major concern for olive groves in Spain and California (USA), where it causes ‘branch and twig dieback’ characterized by wood discoloration, bark canker, and canopy blight. During surveys of olive groves in Apulia (southern Italy), we noticed that—in some areas—trees were heavily affected by severe branch and twig dieback. In addition, chlorosis and the appearance of red-bronze patches on the leaf preceded the wilting of the foliage, with necrotic leaves persisting on the twigs. Given the severity of the manifestation in zones also subject to olive quick decline syndrome (OQDS) caused by *Xylella fastidiosa* subsp. *pauca*, we investigated the etiology and provide indications for differentiating the symptoms from OQDS. Isolation from diseased wood samples revealed a mycete, which was morphologically and molecularly identified as *N. mediterraneum.* The pathogenicity tests clearly showed that this fungus is able to cause the natural symptoms. Therefore, also considering the low number of tested samples, *N. mediterraneum* is a potential causal agent of the observed disease. Specifically, inoculation of the twigs caused complete wilting in two to three weeks, while inoculation at the base of the stem caused severe girdling wedge-shaped cankers. The growth rate of the fungus in in vitro tests was progressively higher from 10 to 30 °C, failing to grow at higher temperatures, but keeping its viability even after prolonged exposure at 50 °C. The capacity of the isolate to produce catenulate chlamydospores, which is novel for the species, highlights the possibility of a new morphological strain within *N. mediterraneum*. Further investigations are ongoing to verify whether additional fungal species are involved in this symptomatology.

## 1. Introduction

The olive tree (*Olea europea* L.) originated from the oleaster 6000–8000 years ago in the northeastern Levant territory of the Mediterranean basin, approximately in the area close to today’s Syria–Turkey border [1]. It was a key crop in early human settlements in the Mediterranean and is still one of the most important and distinctive Mediterranean crops [2]. Its main products—oil and olives—are a typical part of the Mediterranean diet, and along with the leaves helps prevent chronic degenerative and cardiovascular diseases [3,4]. 

As well as being a key feature of the Mediterranean landscape, olive tree cultivation reduces soil erosion—provided that cultivation is not highly intensive and mechanized and is performed in a no-tillage regime, namely with a ground plant cover. In this manner, it enables areas that are not suited to high-income agriculture to be recovered for farming [5]. 

Historically, the soil borne and vascular fungus *Verticillium dahliae* (Kleb.), which is the agent of verticillium wilt, has always been considered as a major threat to olive trees because of its potential for rapid spread, heavy impact on plant viability, and difficulties of applying effective disease management strategies [6,7]. 

Olive quick decline syndrome (OQDS), has recently appeared in the Salento peninsula (Apulia, south-east Italy) causing unprecedented damage to the olive groves. *Xylella fastidiosa* subsp. *pauca* (Schaad, Postnikova, Lacy, Fatmic, and Chang) (Xfp), a quarantine pest, is heavily involved in the etiology. Xfp typically inhabits the xylem, where it multiplies and occludes the vessels, which hampers normal water conduction resulting in rapid canopy scorching and death of the trees [8,9]. There are also several edaphic/climatic factors that might predispose trees to bacterium pathogenicity, thus acting as disease enhancers [10]. 

Interestingly, other decline syndromes of olive trees were reported in the same time span in Italy and other parts of the world which were associated with a complex mycobiota in which some fungal taxa—*Pleurostomophora richardsiae*, Botryosphaeriaceae, *Phaeoacremonium* spp., and Hymenomycetes—seemed to play major roles [11,12,13,14,15].

Since summer 2019, a wilting syndrome affecting olive trees in the provinces of Brindisi and Taranto in Apulia was noted during our surveys and suspected as being different from the Xfp-caused OQDS that was also present in those areas. 

We thus began to investigate the nature of this syndrome by assessing the occur-rence of potentially pathogenic fungi. After running a preliminary sampling, we isolated a botryosphaeriaceous fungus which was identified as *Neofusicoccum mediterraneum* Crous, M.J. Wingf., and A.J.L. Phillips. Since this species has been repeatedly reported in California and Spain as the causal agent of severe twig and branch dieback of olive trees [11,13,15], we retained urgent to report the presence of such dangerous pathogen in Apulia. Thus, we focused this study on the morphological and molecular characterization of this fungus as well as assessing its pathogenicity. 

## 2. Results

### 2.1. Natural Symptoms 

Symptoms that seemed different from those incited by Xfp appeared in summer 2019 and were observed in some olive groves located in the municipalities of Mesagne and San Pietro Vernotico (province of Brindisi) and Lizzano (province of Taranto). The olive groves in Mesagne seemed to be particularly badly affected, with 50% of the trees showing symptoms, and thus we began our investigation there. 

**Figure 1:**
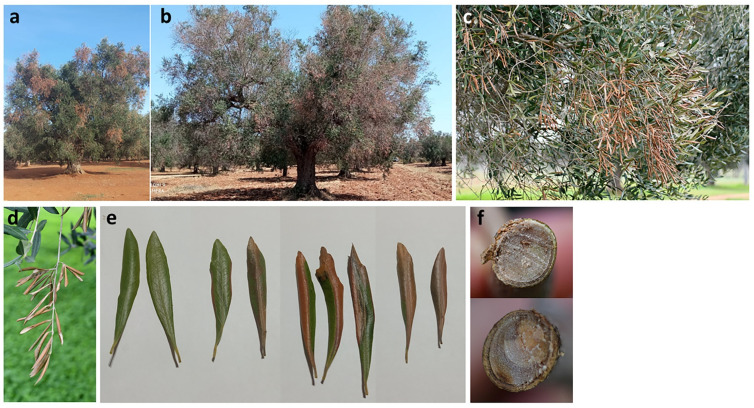
Symptomatic features of branch and twig dieback of olive trees from which *Neofusicoccum mediterraneum* was isolated. (**a**,**b**) overall view; (**c**,**d**) leaf wilting and downward rolling; (**e**) details of symptom progression on leaf blade from chlorosis to appearance of expanding and coalescing red/bronze necrotic lesions; (**f**) wood discoloration in transversal section of branches.

Trees were affected by branch and twig wilting which meant that portions of the canopy appeared scorched (Figure 1a–d). The observation of wilting symptoms at different stages enabled us to establish the symptom progression (Figure 1e): initially red-bronze necrotic patches appeared scattered on the leaf blade or developed from leaf edge or the main vein. Occasionally they were surrounded by a chlorotic halo. Subsequently, the necrotic lesions spread and coalesced, thus affecting the entire leaf blade which rolled downward. In other cases, a generalized chlorosis of foliage preceded rolling and wilting. At the base of the wilted portion of the branches, there were points in which the transversal section of the vascular tissue—1 to 3 cm in diameters—appeared discolored, suggesting a dysfunction in water conduction leading to water leaking from leaf blades not compensated for by an adequate transport in the xylem vessels (Figure 1f). Externally, the bark showed sunken and reddish areas. 

**Figure 2:**
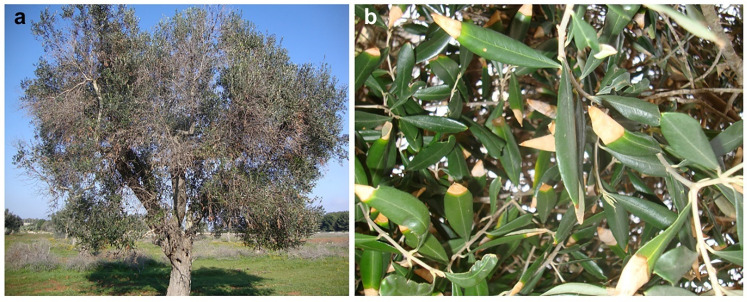
Symptomatic features of olive quick decline syndrome (OQDS) caused by *Xylella fastidiosa* subsp. pauca (Xfp). (**a**) Overall view; (**b**) desiccation starting from the leaf tip (though frequently occurring, leaf tip desiccation is not pathognomonic for OQDS).

The symptoms observed are distinct from those caused by Xfp as reddening of leaf blade and wood discoloration are not observed in Xfp-infected trees which show aspecific canopy scorching and desiccation of leaf tips (Figure 1 and Figure 2).

### 2.2. Assessment of Fungi and Bacteria in Twigs and Branches Symptomatic Samples

Isolations yielded a number of fungal isolates which were identified at the genus level as *Pseudocamarosporium* sp. (from five trees out of eight), *Paraconiothirium* sp. (from two trees). Botryosphaeriaceous isolates characterized by dark grey and fast-growing mycelia were isolated from four of the sampled trees. While we provisionally assigned these isolates to the genus *Neofusicoccum* (three isolates from three trees) and *Diplodia* (three isolates from three trees), we also assessed their virulence degree in preliminary trials (unpublished data). Given that the *Neofusicoccum*-like isolate CREA-DC TPR OL.427 expressed the highest virulence we preferred to focus this study on this isolate. The other undetermined *Neofusicoccum*-like isolates were different from CREA-DC TPR OL.427 (unpublished data). Characterization of the Botryosphaeraceae other than CREA-DC TPR OL.427—identification and pathogenicity—is ongoing.

Isolation and real-time PCR ruled out the presence of *V. dahliae*. Xfp was detected in three sampled trees, but the one infected with the botryosphaeriaceous isolate CREA-DC TPR OL.427, object of this study, was negative. 

### 2.3. Morpho-Cultural Characterization of the Botryosphaeriaceous Isolate CREA-DC TPR OL 427

**Figure 3:**
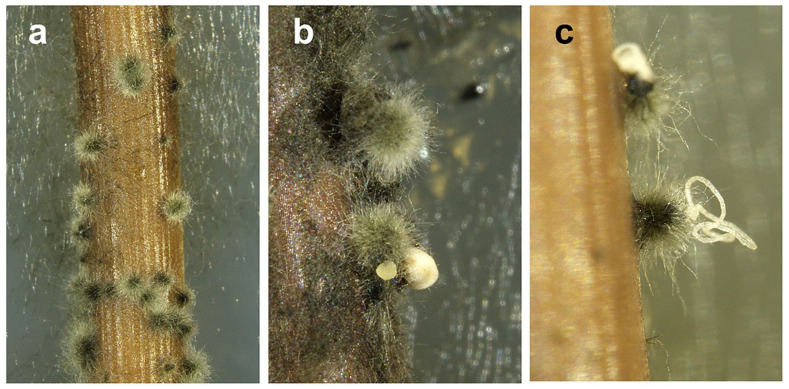
Typical hairy conidiomata of *Neofusicoccum mediterraneum* from Apulia (Italy) developed on pine needles in water agar. In (**a**) immature conidiomata, in (**b**,**c**) mature conidiomata exuding globose or filiform masses of conidia.

**Figure 4:**
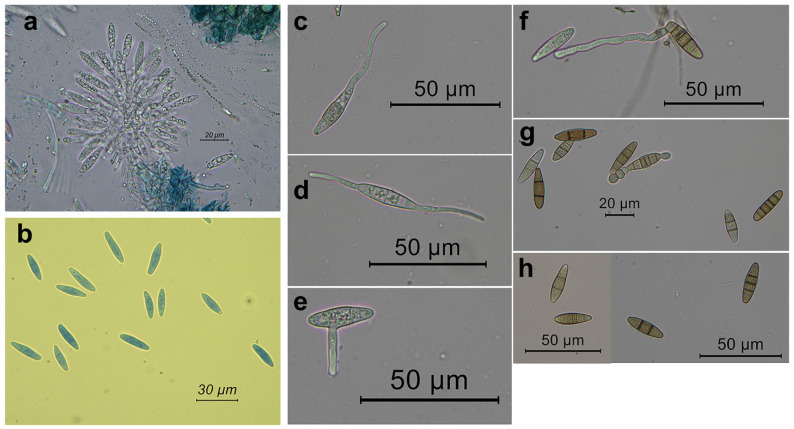
Conidiogenous cells and conidia from conidiomata of *Neofusicoccum mediterraneum* from Apulia (Italy), developed on pine needles. In (**a**) a tuft of conidiogenous cells from the internal of a conidiomata; (**b**) conidia; (**c**–**f**) germination patterns of the conidia; (**f**–**h**) conidia with a variable number of septa.

After five days of culturing on pine needle agar, the botryosphaeriaceous isolate CREA-DC TPR OL.427 began to produce globose hairy conidiomata on pine needles. These conidiomata began to exude mucoid masses of conidia roughly 7–10 days after culturing (Figure 3). Tufts of subcylindrical/fusoid-ellipsoidal conidiogenous cells were observed within the conidiomata. Conidia were devoid of any persistent mucous sheath; they were hyaline, smooth, thin-walled, fusoid-ellipsoidal, and were widest in the middle or in the upper third, apex subobtuse, base subtruncate, 0–5 transversally-septate (Figure 4). We measured conidia within conidiomata and when exuded outside. Within conidiomata the size of conidia was: 19.5–30.2 μm (mean = 23.6, SD = 1.8) × 4.5–7.1 μm (mean = 6.1, SD = 2.1), length/width = 3.9, (*n* = 146); in the exuded mass the size was: 19.6–33.8 μm (M = 24.1, SD = 2.1) × 5–7.5 μm (M = 5.9, SD = 0.4), length/width = 4.1, (*n* = 146).

Germination was induced by incubating conidia in water. Germ tubes emerged from one or both the apical ends, from the lateral of one apical end or along the conidium side (Figure 4).

On the basis of these features and using the identification key to genera and species of Botryosphaeriaceae by Phillips et al. [16], we identified the botryosphaeriaceous fungus as *Neofusicoccum mediterraneum* (Ascomycota; Pezizomycotina; Dothideomycetes; Dothideomycetes incertae sedis; Botryosphaeriales; Botryosphaeriaceae; Neofusicoccum).

**Figure 5:**
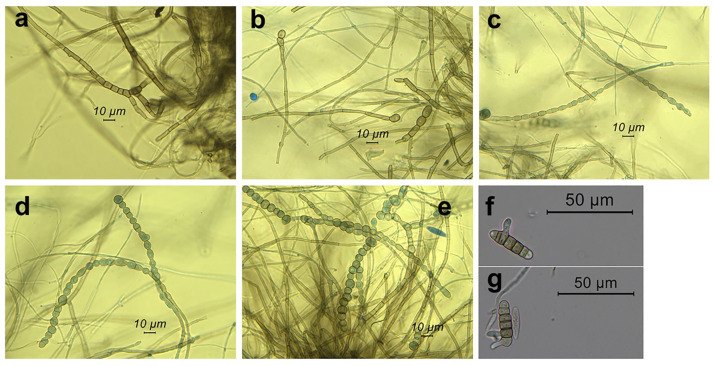
Chlamydospores of *Neofusicoccum mediterraneum* from Apulia (Italy) grown on pine needles. In a → e catenulate chlamydospores from conidiomata. In (**a**) formation of increasingly close septa in the iphae which precedes chlamydospore formation; (**b**) formation of chlamydospores terminal to the iphae; (**c**–**e**) chains of chlamydospores; (**f**,**g**) conidia giving rise to chlamydospores after septa formation.

We also found an additional morphological feature that was not included in the steps of the key to the Botryosphaeriaceae [16]: frequently inside the conidiomata and more rarely in the mycelium of the axenic culture, we observed chains of globose to subglobose, ellipsoid, oval, or subcylindrical, dark-brown chlamydospore-like bodies. They were intercalary and even terminal to the iphae. Formation of increasingly close septa in the iphae and the rounding off of the single cells gave rise to the chlamydospore-like bodies. The chains ended up thus releasing free single bodies (Figure 5). Interestingly, conidia also gave rise to chlamydospore-like bodies after forming multiple septa (Figure 5). Size was: 5.4–11.5 μm (M = 7.8, SD = 1.3) × 5.4–9.9 μm (M = 7.1, SD = 0.9), (*n* = 35). 

**Figure 6:**
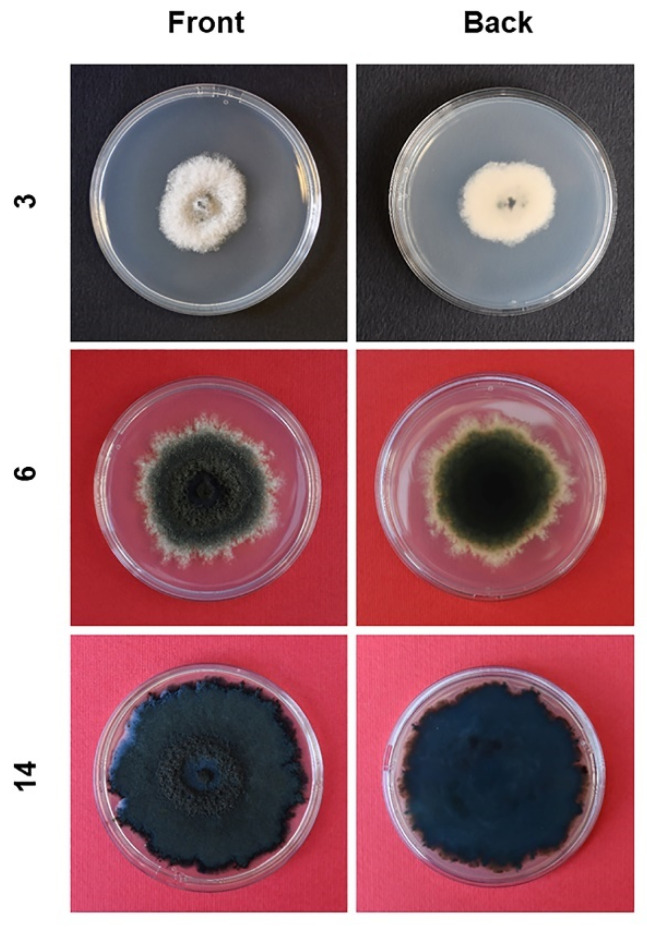
Morphological evolution of *Neofusicoccum mediterraneum* isolated from olive trees in Apulia (Italy) and grown as an axenic culture on PDA. Lateral numbers indicate the age of the cultures in days.

Colonies grown in axenic culture were white and fluffy in the first three days of growth then becoming iron-grey/black with a dark bluish tonality; two-week-old colonies formed a dense flat mycelium with a fluffy central circular band (Figure 6).

**Figure 7:**
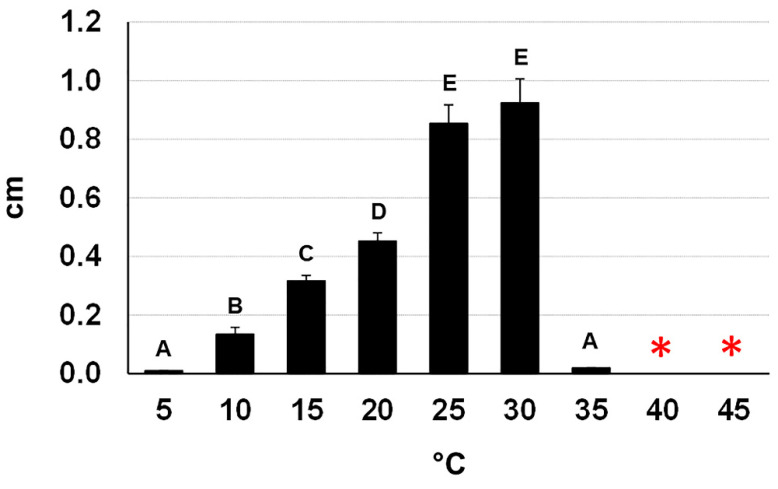
Daily growth rates of *Neofusicoccum mediterraneum* (from Apulia, Italy) on PDA at different temperatures. Different letters indicate statistically significant differences among the treatments (*p* < 0.01). The asterisks mean that the fungus remained viable after a five-day exposure at the reported temperatures.

The fungus was able to grow at 10–30 °C with a peak at 30 °C—confirming what Moral et al. reported [11] and to remain viable after a five-day exposure up to 45 °C (Figure 7). Interestingly, incubation at 50 °C of mycelial plugs taken from five-day-old cultures completely compromised their viability after incubation for 18 h and nearly completely for six hours (one plug viable out of 15). In contrast, all the mycelial plugs taken from 20-day-old cultures had a full survival up to 18 h of incubation and a nearly full survival after 24 h (1 plug devitalized out of 15). We did not test longer incubation times because, at 50 °C, the PDA dehydrated and split. 

### 2.4. Sequencing and Phylogenetic Analysis for Species Identification of the Selected Isolate 

In an NCBI Blastn search, ITS, TEF1-α, and TUB2 sequences from the botryosphaeriaceous isolate obtained with isolation, aligned with 100% nucleotide identity with strains of *Neofusicoccum mediterraneum* (subjects: MG745841 for ITS, KX029218 for TEF1-α and GU251835 for TUB2). 

**Figure 8:**
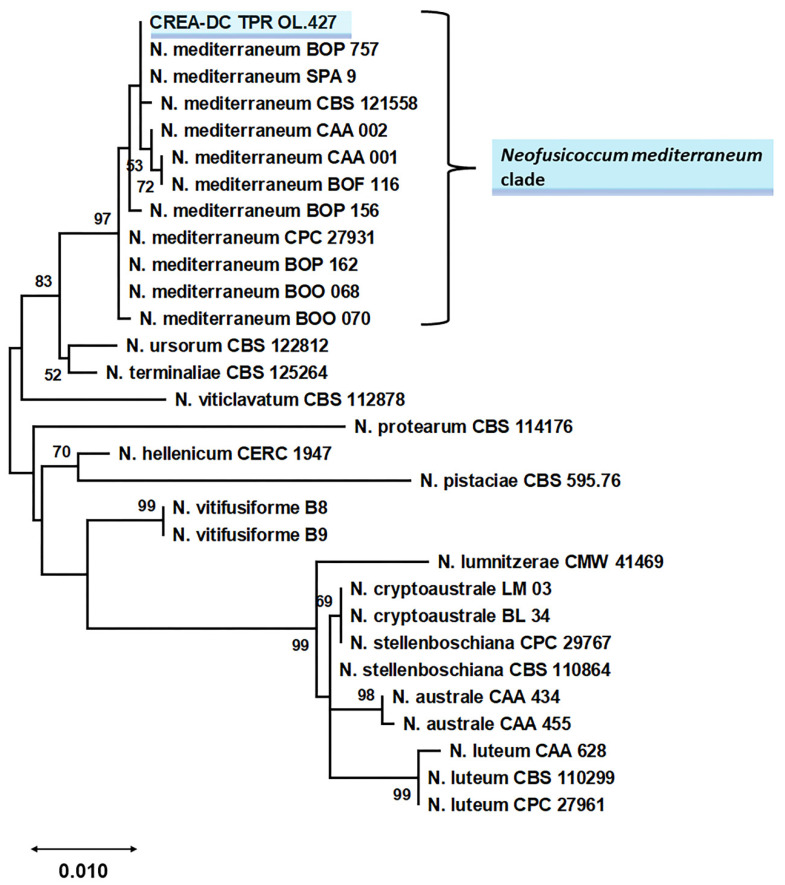
Phylogeny of *Neofusicoccum* genus, based on ITS + TUB2 + TEF1-α data set [11,17,18] and including the botryosphaeriaceous isolate from Apulia (Italy), CREA-DC TPR OL.427 (shaded in blue-sky). The phylogeny was inferred using the ML method and general time reversible model [19], with 1000 bootstraps. The tree with the highest log likelihood (−4454.50) is shown. The percentage of trees in which the associated taxa clustered together is shown next to the branches. Bootstrap values lower than 50 are not reported. The tree is drawn to scale, with branch lengths measured in the number of substitutions per site. This analysis involved 61 nucleotide sequences. There was a total of 1150 positions in the final dataset. For the sake of legibility, we show a subtree containing the *Neofusicoccum mediterraneum* clade. In Figure S1, the entire phylogenetic tree is shown.

Phylogenetic relationships of *Neofusicoccum* species fully matched the topology of the *Neofusicoccum*-specific phylogeny reported by Lopes et al. [17] and Bezzera et al. [18]. Sequence data of the isolate from Apulia grouped with the maximum bootstrap value with reference *N. mediterraneum* (Figure 8) thus confirming the morphological identification. 

### 2.5. Pathogenicity Tests

The twigs inoculated on 17 May and on 16 June 2021 were all evidently wilted after 23 and 14 days, respectively. Long necrosis streaks affected the bark as well as the xylem, progressing upward and downward of the inoculation point (Table 1). The twigs used as control kept leaf turgor and growth vigor.

**Figure 9:**
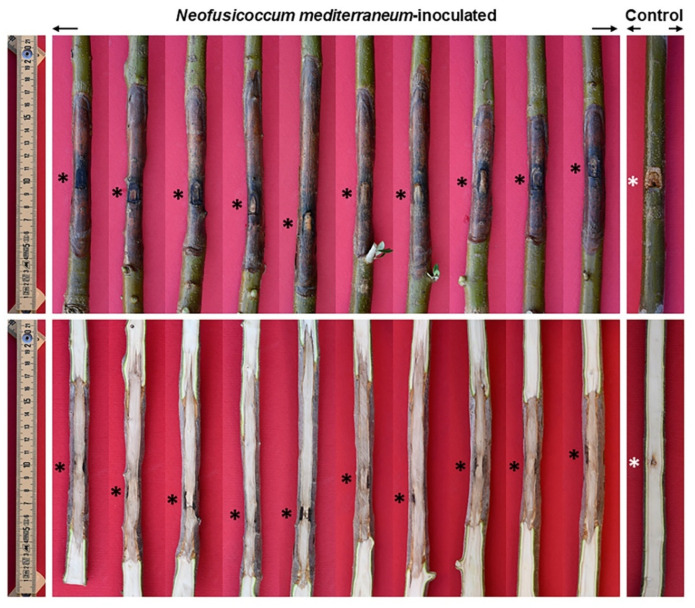
Bark necrosis (above) and wood discoloration (below) at the stem of three-year-old olive trees inoculated with *Neofusicoccum mediterraneum*. Asterisks indicate the inoculation points.

**Figure 10:**
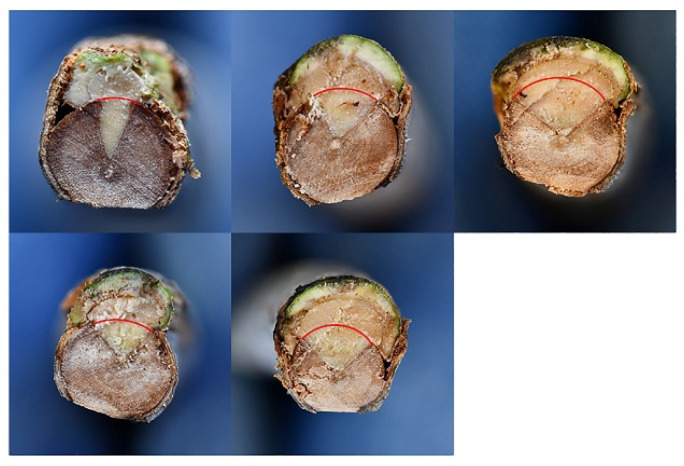
Girdling necrosis and its progression in the xylem according to a wedge-shaped model (transversal section) following inoculation at the stem of three-year-old olive trees inoculated with *Neofusicoccum mediterraneum*. Red lines define the original circumference of the stem before the inoculation; the healthy tissue above is a neoformation which originated as the host reaction to fungal infection.

Fungal inoculation at the basal portion of the stem (17 June 2021) caused severe bark canker progressing upward and downward from the point of inoculation (Figure 9). The length of bark canker was 12.2 cm on average (SD = 2.7) and the corresponding internal discoloration was 20.3 cm on average (SD 8.7). Bark necrosis girdled the stem circumference almost entirely. The girdling index was between 0.6 and 1. In the transversal section the progression of wood discoloration was wedge-shaped, outside-in, and affected much of the transversal section (Figure 10). The inoculation wounds of plants used as controls completely healed and showed a minimal discoloration in the wood behind it.

*N. mediterraneum* was reisolated from the spreading necrosis of all the inoculated plants, but not from healthy control plants. Koch’s postulate was thus fulfilled.

## 3. Discussion

The decline of olive trees in terms of twig and branch dieback (canopy blight, bark canker, and wood discoloration) caused by fungal pathogens other than *V. dahliae* has been overlooked worldwide as it is easily confused with verticillium wilt [13]. Similarly, after the OQDS outbreak, an additional symptom misinterpretation tended to emerge as any olive dieback in Apulia was suspected of being OQDS. To clarify this matter, from 2010 onwards extensive investigations demystified the etiology of these symptoms highlighting the occurrence of additional fungal agents that were a threat to the longevity and viability of olive trees [11,12,13,14,15]. The matter and the list of fungal species involved are reviewed by Moral et al. [15]. Of these fungi, Botryosphaeriaceae seem the most important.

Botryosphaeriaceae infect a wide range of perennial plant species both in agricultural plantations and in forest ecosystems in which a latent phase alternates with full virulence according to the degree of stress suffered by the host [20]. 

Several species in the Botryosphaeriaceae family have been associated with dieback of olive trees and for which pathogenicity tests have revealed varying degrees of virulence. *Diplodia seriata* was reported as an aggressive agent of olive dieback in Croatia [21]. *D. mutila* was shown to be one of the most virulent agents of olive dieback in California, while in the same study *N. luteum* showed moderate virulence [13]. *N. parvum* has been shown to be a key agent in the dieback of olive and oleaster trees, together with other fungal species [12,22]. *N. mediterraneum* has been reported in olive trees affected by severe twig and branch dieback in California and Spain, and in comparative pathogenicity tests it was shown to be more aggressive than the other botryosphaeriaceous species [11,13,15,23]. Interestingly, *N. mediterraneum* has also been reported in Apulia in olive trees, but only in the drupes and with a low occurrence (less than 1% of the examined drupes) [24]. Other botryosphaeriaceous species have shown low or no virulence in olive trees, though they were associated in the field with severe decline, such as: *Lasiodiplodia theobromae, Dothiorella iberica*, *Botryosphaeria dothidea*, and *N. vitifusiforme* [13,15]. 

In our study, the *N. mediterraneum* isolate recovered in Apulia was clearly shown to be involved in the observed disease that we named branch and twig dieback, in line with Moral et al. [11,15] and Úrbez-Torres et al. [13]. Although we have yet to thoroughly evaluate the association of *N. mediterraneum* with the disease and, possibly, identify additional agents, we believe that it is important to report the occurrence of this dangerous pathogen now, due to the severity of the natural symptoms, which were fully confirmed by our pathogenicity tests. 

In fact, in this study, *Neofusicoccum mediterraneum* was revealed to be highly virulent as it killed olive twigs within a time-span of 2–3 weeks and caused severe girdling and wedge-shaped cankers at the base of the stem of three-year-old trees. The fact that reddening of the leaf blades was not observed in our inoculation trials, as it was in the field, could be due to the extremely rapid wilting which affected the twigs and, regarding the plants inoculated at the stem, longer observation times might have been necessary for this foliar symptom to appear. 

Interestingly the pathogenicity tests were conducted in late spring/summer 2021 which was characterized by very high temperatures (Figure S2). In line with the results of in planta trials, the in vitro growth curve of the *N. mediterraneum* isolate showed that the fungus is thermophilic and thermotolerant as it can even survive prolonged exposure at 50 °C. This suggests that *N. mediterraneum* is an increasingly emerging pathogen in an environment that is subject to warming temperatures.

The specific nature of the *N. mediterraneum* isolate under study was clearly assessed by multi-locus phylogenetic analysis and confirmed by the morphological key by Phillips et al. [16]. Interestingly, the fungus produced catenulate chlamydospores, a well-known feature of several botryosphaeriaceous genera including *Neofusicoccum*, but not reported to date for *N. mediterraneum* [16,25]. It will be interesting in the future to verify whether the Apulian population of *N. mediterraneum* is characterized by this feature, which would establish a new morphological/geographical strain within this fungal species.

Additional in-depth surveys in the area will be necessary in order to assess the diffusion of the disease, without neglecting the possible involvement of additional Botryosphaeriaceae. In this same study we also assessed the occurence of undetermined botryosphaeriaceous species other than *N. mediterraneum*. Thus, the possibility of a multiple involvement by Botryospaeriaceae is real and worth considering. In fact, several Botryosphaeriaceae have been found associated not only with branch and twig dieback of olive trees [13], but also linked to a decline of *Eucalyptus camaldulensis* characterized by branch and twig dieback—namely bark cankers, wedge-shaped wood necrosis, and death of portions of canopy. Specifically, five *Neofusicoccum* species were found in the declining trees, all of which were pathogenic to *E. camaldulensis*, but *N. mediterraneum* was the most aggressive albeit not the most frequent [26]. Similarly, it is well known that several Botryosphaeriaceae species are involved in “Botryosphaeria dieback” of grapevine [27]. *Citrus* plants have also been reported to be heavily damaged by various Botryosphaeriaceae species throughout Europe [18].

It is also worth mentioning that a dieback of olive trees was investigated in Apulia, reported in 2013, and attributed primarily to *Pleurostomophora richardsiae* and secondarily to *Neofusicoccum parvum* [12]. However, the disease we report in this paper is different. In fact, we did not detect the above cited fungi and on the other side *N. mediterraneum* was not revealed in that investigation, nor were some typical symptomatological features here described—i.e., leaf blade reddening and rolling. 

Although OQDS caused by Xfp is widespread in southern Apulia and both OQDS and branch and twig dieback are characterized by severe wilting, they can be differentiated upon careful observation: (a) OQDS is not associated with wood discoloration which is a typical internal feature of the branch and twig dieback observed in Apulia; (b) OQDS canopy scorching is not preceded by the reddening of leaf blade, which is typical of the branch and twig dieback observed in Apulia; (c) OQDS leaf scorching may be preceded by the necrosis of leaf tips that progressively extends toward the petiole, a symptom not specific of Xfp infections, but which was not observed in the branch and twig dieback in Apulia. See Frisullo et al. for symptoms of OQDS [9].

## 4. Materials and Methods

### 4.1. Symptom Survey, Sample Collection, Isolations, and Detections

The survey was carried out in December 2019 in an olive orchard with trees older than 150 years, cv Ogliarola salentina and Cellina di Nardò, spaced at 20 × 15 mt, and located in the municipality of Mesagne (Brindisi province, Apulia, southern Italy). 

Small branches and twigs of eight diseased trees were sampled. Alterations to the internal tissue were explored by sectioning. Isolations were performed from the discoloured/necrotic wood matching fresh reaction zones. From these areas small fragments (size ≤ 1 mm) were dissected, rapidly burned, and plated on two types of cultural media: potato dextrose agar (PDA) (Oxoid, Thermo Fisher Scientific, Waltham, MA, USA) with 300 mg L^−1^ streptomycin with or without benomyl [Methyl 1-(butylcarbamoyl)-2-benzimidazolecarbamate] (Merck KGaA, Darmstadt, Germany). The benomyl-containing medium was used in order to select Basidiomycota, if present. Thirty-six fragments were plated on each medium, thus 72 for each sample tree. All plates were incubated at 25 °C, in the dark. Fungal isolates were selected by transferring minute mycelial fragments from the advancing edges of the colonies on fresh PDA plates and conserved at 5 °C in PDA slant tubes.

In parallel, samples were also tested with Xfp and *V. dahliae*-targeted Real-Time PCR procedures [28,29,30]. For Xfp detection, a multiple sampling was performed for each tree, as previously reported [31]. 

### 4.2. Morphological Features of the Selected Fungal Isolate and Its Cultural Characteristics 

Among the isolated fungi we focused on a single botryosphaeriaceous isolate (iron-grey/black fluffy mycelium) (CREA-DC TPR OL.427). Conidiomata formation and sporulation was induced on 2% water agar (WA) (Oxoid) with autoclaved *Pinus pinea* needles as the substratum, at 23 °C under near-UV light [32]. Conidia germination was induced in an aqueous suspension, in 48–72 h, at room temperature. Fungal structures were observed under a light microscope—a Leica DM6B, equipped with a Leica LAS X software—in order to record magnified depictions, and Leica DFC 7000T camera for image acquisition. 

Colonies grown on PDA plates (90 mm in diameter) were observed and photographed at different times in order to document age-related changes.

To determine the optimal growth temperature and thermal viability of the isolate, the growth rate was assessed at temperatures from 5 to 45 °C with a step-size of five, in the dark. For each temperature, five PDA plates, 90 mm in diameter, were inoculated with a fungal plug 9 mm in diameter taken from an actively growing colony. The diameter of each colony was measured twice at right angles after the colony had covered roughly 70% of the surface for the fast-growing rates (20, 25, 30 °C), or after 10 days (5, 10, 15 °C) and 5 days in cases where no growth occurred at the highest temperatures (35, 40, and 45 °C). In the latter case, the viability of the mycelium was tested by transferring the inoculated plates at 25 °C. Growth data were used to infer the medium daily growth.

Fungal viability was also tested at 50 °C: plugs were collected from actively expanding colonies (5 days old) and from mature colonies (20 days old) and incubated at 50 °C in the dark for 6, 18, and 24 h. A total of 15 plugs were used per treatment (temperature/colony age), which had been cultured on three PDA plates 90 mm in diameter. Viability was checked as described above. 

### 4.3. Sequencing and Phylogenetic Analysis for Species Identification 

The botryosphaeriaceous isolate (CREA-DC TPR OL.427) was subjected to a multi-locus sequencing. After extracting fungal gDNA from an axenic culture of the isolate under study, the complete ITS region, beta-tubulin 2 (TUB2) and translation elongation factor 1-alpha (TEF1-α) were amplified and sequenced to find matches in the NCBI GenBank and to infer a multi-locus phylogeny for species determination. Details on fungal gDNA extraction, primers, reaction assembly, and thermal cycling of the amplifications are reported in Text/Table S1 [33,34,35,36].

PCR amplicons were directly sequenced in both directions by Sanger technology (Bio-Fab research s.r.l. Rome Italy). Sequences newly generated in this study were deposited in the NCBI GenBank with Acc Nos OL454501, OL539661, and OL539662.

Sequences are included in Text/Table S1. They were compared with GenBank accessions using the blastn suite on the NCBI server (https://blast.ncbi.nlm.nih.gov/Blast.cgi?LINK_LOC=blasthome&PAGE_TYPE=BlastSearch&PROGRAM=blastn) (access on 1 October 2021). 

Based on the blast results, a *Neofusicoccum*-specific phylogeny was inferred to confirm the specific nature of the isolate under study. A combined multiloci dataset was used as input for the analysis, ITS + TUB2 + TEF1-α. The reference sequences were those used by Lopes et al. [17], Bezerra et al. [18] plus others found in NCBI GenBank. All strains lacking at least one of the three required DNA sequences were discarded. *Botryosphaeria dothidea* was included as the outgroup (Spreadsheet S1) [18]. Sequences of the isolate under study were aligned to reference sequences, each locus separately, with MAFFT on the EMBL-EBI server (htpps://www.ebi.ac.uk) (access on 1 November 2021). The alignments were then trimmed to span the same region and concatenated in MEGA X [37]. The evolutionary history was inferred by using the maximum likelihood (ML) method and general time reversible model [19]. Bootstrap values were calculated from 1000 replicates. 

Initial tree(s) for the heuristic search were obtained automatically by applying neighbor-join and BioNJ algorithms to a matrix of pairwise distances estimated using the maximum composite likelihood (MCL) approach, and then selecting the topology with superior log likelihood value. A discrete Gamma distribution was used to model evolutionary rate differences among sites (5 categories (+*G*, parameter = 0.2528)). Evolutionary analyses were conducted in MEGA X [37].

### 4.4. Pathogenicity Tests

Inoculation trials were carried out to assess the pathogenicity of the *Neofusicoccum*-like isolate under study (CREA-DC TPR OL.427). 

Olive trees (cv. Frantoio) were supplied by Spoolivi - Società Pesciatina d’Olivicoltura (Pescia, PT, Tuscany, Italy), transplanted into 23-liter pots and used for the inoculation trials. The pot substrate was Radicom (Vigorplant, Lodi, Lombardy, Italy), containing a mixture of peat moss, black peat, marsh peat, and vegetable compost. Humus was present in the form of humic and fulvic acids with a water pH of 6–6.5. 

Three-year-old olive trees at third-year-age were used for one-year-old twig inoculation, whereas those at fourth-year-age were used for inoculation of the basal portion of the stem, at a height of 15–20 cm. The inoculations were performed as follows: rectangular PDA-mycelium plugs (16 × 3–4 mm sized for twig, and 20 × 8–10 mm for stem inoculation) were cut from an actively growing colony and placed on similarly sized wounds—i.e., on the xylem surface which had been exposed by cutting the bark top-down with a razor blade through the cambium. The bark strip was then gently set on the plug. The inoculation point was covered with a cotton disk wetted with 3 ml of sterile water, and wrapped with a sterile strip of aluminium foil, which was taped to the stem at the top and bottom edges. The cover was removed after 25–30 days. 

For each trial, we used 10 plant replicates for fungal inoculation and five for inoculation with sterile PDA (the negative control). With regard to the twig trial, we inoculated two twigs for each plant replicate both in the fungal and control treatments, thus providing 20 replicates for the fungal-inoculated plot and 10 for the control.

The status of plants was monitored until full symptom expression or until death of the inoculated parts. Given the short duration of the test, twig inoculation was performed twice, on 17 May 2021 and on 16 June 2021. Stem inoculation was performed on 17 June 2021 and observed for four months.

At the end of the trials, the length of the external bark necrosis/canker and internal wood discoloration was recorded. The length of the external healing reactions and of the corresponding wood discolouration behind them was also recorded in the control plants. In the stem-inoculated plants, we calculated the ratio between the tangential spread of bark necrosis and the stem circumference, which we define here as the girdling index. Thus, this index ranges from 0 to 1 (1 means that necrosis completely girdles the stem) Fungal re-isolation was performed on streptomycin-supplemented PDA plates.

### 4.5. Statistical Analyses 

A one-way fully randomized analysis of variance (ANOVA or Welch test) and the Tukey test as post-hoc analysis, were carried out to compare the fungal growth at different temperatures. PAST version 4.03 was used for the analysis [38]. 

## 5. Conclusions

The symptomatology—branch and twig dieback—that is emerging with severity in Apulia adds further worry for olive-growing already damaged by OQDS.

*Neofusicoccum mediterraneum*, isolated from a single diseased tree, was fully able to cause the disease symptoms in the pathogenicity trials, thus showing a clear potential to be a causal agent of the disease. However, to date, informations on the diffusion of this pathogen in the area affected by the disease are lacking. Thus, more research is needed to consolidate our results.

We consider this study as a first step paving the way for future in depth investigations in the Apulian area shedding more light on a possibly complex etiology and the epidemiology. Moreover, estimating the overlap between OQDS and branch and twig dieback caused by *N. mediterraneum*, and possibly other botryosphaeriaceous species, will also be crucial even in the light of the development of effective control strategies. 

## Figures and Tables

**Table 1:** Trials to test the pathogenicity of *N. mediterraneum* and symptoms reproduction.

Twig Trials	Replicates	Ø ^1^ (cm)	Bark Canker cm (SD)	Wood Discoloration cm (SD)	Wilting ^2^
17 May 2021	20	0.35–0.5	7.1 (1.8)	16.9 (8.1)	+
16 June 2021	20	0.45–0.7	7.0 (2.6)	9.1 (2.4)	+
Stem trial					
17 June 2021	10	1.35–1.55	12.2 (2.7)	20.3 (8.7)	-

^1^ Ø = range of diameters of the twig/stem at the inoculation point; ^2^ All replicate within each trial gave the same response: + wilting, - normal vegetative condition.

## Data Availability

All data regarding this study have been submitted.

## Supplementary Materials

The following are available online at https://www.mdpi.com/article/10.3390/pathogens11010053/s1, Text/Table S1. Methods for DNA extraction, PCR amplifications and sequencing of DNA regions of *Neofusicoccum mediterraneum* isolated from olive trees in Apulia (Italy). Table S1A reports the DNA regions amplified and primers used, and Table S1B reports the thermal cycling for each DNA region. The sequences generated in this study and their accession numbers are listed below. Spreadsheet S1. Reference sequences used to infer the multi-locus phylogeny of the *Neofusicoccum* genus. Figure S1. Growth percentage of *Neofusicoccum mediterraneum* isolated from olive trees in Apulia (Italy) on PDA supplemented with the agrochemicals compared with growth on PDA non supplemented (=100%). Statistical analysis was performed on arcsine transformed percentage values. Different letters indicate significant differences from each other (*p* < 0.05). Figure S2. Maximum (red) minimum (azure) and average (grey) temperatures during the pathogenicity tests (inoculations on twigs were on 17 May and 16 June 2021, inoculations on stems were on 17 June 2021).
