# Supplementary material for: Neofusicoccum mediterraneum Is Involved in a Twig and Branch Dieback of Olive Trees Observed in Salento (Apulia, Italy)
Source: Pathogens. 2022 Jan 2;11(1):53. doi: 10.3390/pathogens11010053 (PMC8780778; doi:10.3390/pathogens11010053)
Supplement: Supplementary file 1 [file pathogens-11-00053-s001.zip › Figure S1.pdf]

Title:
Twig and branch dieback of olive trees in Salento (Apulia, Italy) caused by *Neofusicoccum mediterraneum*

Journal name:
Pathogens - MDPI

Authors:
Angela Brunetti, Antonio Matere, Valentina Lumia, Vittorio Pasciuta, Valeria Fusco, Domenico Sansone, Paolo Marangi, Nicola Cristella, Francesco Faggioli, Marco Scortichini and Massimo Pilotti

Corresponding author:
Massimo Pilotti

Affiliation:
Institution: Council for Agricultural Research and Economics; Department: Research Center for Plant Protection and Certification (CREA-DC) - Via C. G. Bertero 22, 00156 Rome, Italy

E-mail address:
massimo.pilotti@crea.gov.it

Figure S1 Phylogeny of *Neofusicoccum* genus, based on ITS + TUB2 + TEF1-α data set [11, 17, 18] and including the botryosphaeriaceous isolate from Apulia (Italy), CREA-DC TPR OL.427 (shaded in blue-sky). The phylogeny was inferred using the ML method and General Time Reversible model [19], with 1000 bootstraps. The tree with the highest log likelihood (-4454.50) is shown. The percentage of trees in which the associated taxa clustered together is shown next to the branches. Bootstrap values lower than 50 are not reported. The tree is drawn to scale, with branch lengths measured in the number of substitutions per site. This analysis involved 61 nucleotide sequences. There was a total of 1150 positions in the final dataset.

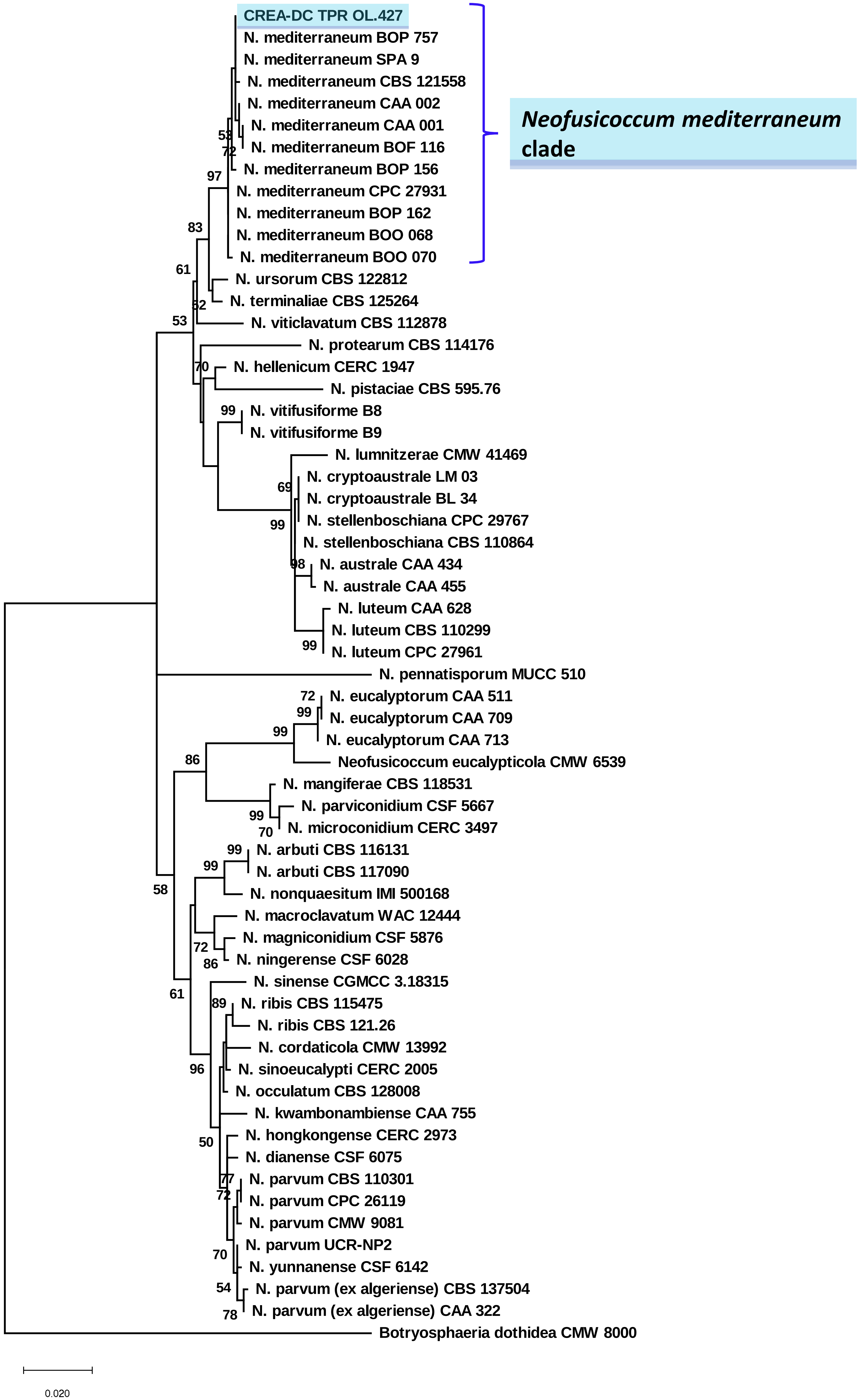
CREA-DC TPR OL.427
N. mediterraneum BOP 757
N. mediterraneum SPA 9
N. mediterraneum CBS 121558
N. mediterraneum CAA 002
N. mediterraneum CAA 001
N. mediterraneum BOF 116
N. mediterraneum BOP 156
N. mediterraneum CPC 27931
N. mediterraneum BOP 162
N. mediterraneum BOO 068
N. mediterraneum BOO 070
Neofusicoccum mediterraneum clade
N. ursorum CBS 122812
N. terminaliae CBS 125264
N. viticlavatum CBS 112878
N. protearum CBS 114176
N. hellenicum CERC 1947
N. pistaciae CBS 595.76
N. vitifusiforme B8
N. vitifusiforme B9
N. lumnitzerae CMW 41469
N. cryptoaustrale LM 03
N. cryptoaustrale BL 34
N. stellenboschiana CPC 29767
N. stellenboschiana CBS 110864
N. australe CAA 434
N. australe CAA 455
N. luteum CAA 628
N. luteum CBS 110299
N. luteum CPC 27961
N. pennatisporum MUCC 510
N. eucalyptorum CAA 511
N. eucalyptorum CAA 709
N. eucalyptorum CAA 713
Neofusicoccum eucalypticola CMW 6539
N. mangiferae CBS 118531
N. parviconidium CSF 5667
N. microconidium CERC 3497
N. arbuti CBS 116131
N. arbuti CBS 117090
N. nonquaesitum IMI 500168
N. macroclavatum WAC 12444
N. magniconidium CSF 5876
N. ningerense CSF 6028
N. sinense CGMCC 3.18315
N. ribis CBS 115475
N. ribis CBS 121.26
N. cordaticola CMW 13992
N. sinoeucalypti CERC 2005
N. occulatum CBS 128008
N. kwambonambiense CAA 755
N. hongkongense CERC 2973
N. dianense CSF 6075
N. parvum CBS 110301
N. parvum CPC 26119
N. parvum CMW 9081
N. parvum UCR-NP2
N. yunnanense CSF 6142
N. parvum (ex algeriense) CBS 137504
N. parvum (ex algeriense) CAA 322
Botryosphaeria dothidea CMW 8000
0.020
