# Supplementary material for: Neofusicoccum mediterraneum Is Involved in a Twig and Branch Dieback of Olive Trees Observed in Salento (Apulia, Italy)
Source: Pathogens. 2022 Jan 2;11(1):53. doi: 10.3390/pathogens11010053 (PMC8780778; doi:10.3390/pathogens11010053)
Supplement: Supplementary file 1 [file pathogens-11-00053-s001.zip › Figure S2.pdf]

Title:
Twig and branch dieback of olive trees in Salento (Apulia, Italy) caused by *Neofusicoccum mediterraneum*

Journal name:
Pathogens - MDPI

Authors:
Angela Brunetti, Antonio Matere, Valentina Lumia, Vittorio Pasciuta, Valeria Fusco, Domenico Sansone, Paolo Marangi, Nicola Cristella, Francesco Faggioli, Marco Scortichini and Massimo Pilotti

Corresponding author:
Massimo Pilotti

Affiliation:
Institution: Council for Agricultural Research and Economics; Department: Research Center for Plant Protection and Certification (CREA-DC) - Via C. G. Bertero 22, 00156 Rome, Italy

E-mail address:
massimo.pilotti@crea.gov.it

Figure S2 Maximum (red) minimum (azure) and average (grey) temperatures occurred during the pathogenicity tests (inoculations on twigs were on 17 May and 16 June 2021, inoculations on stems were on 17 June 2021)

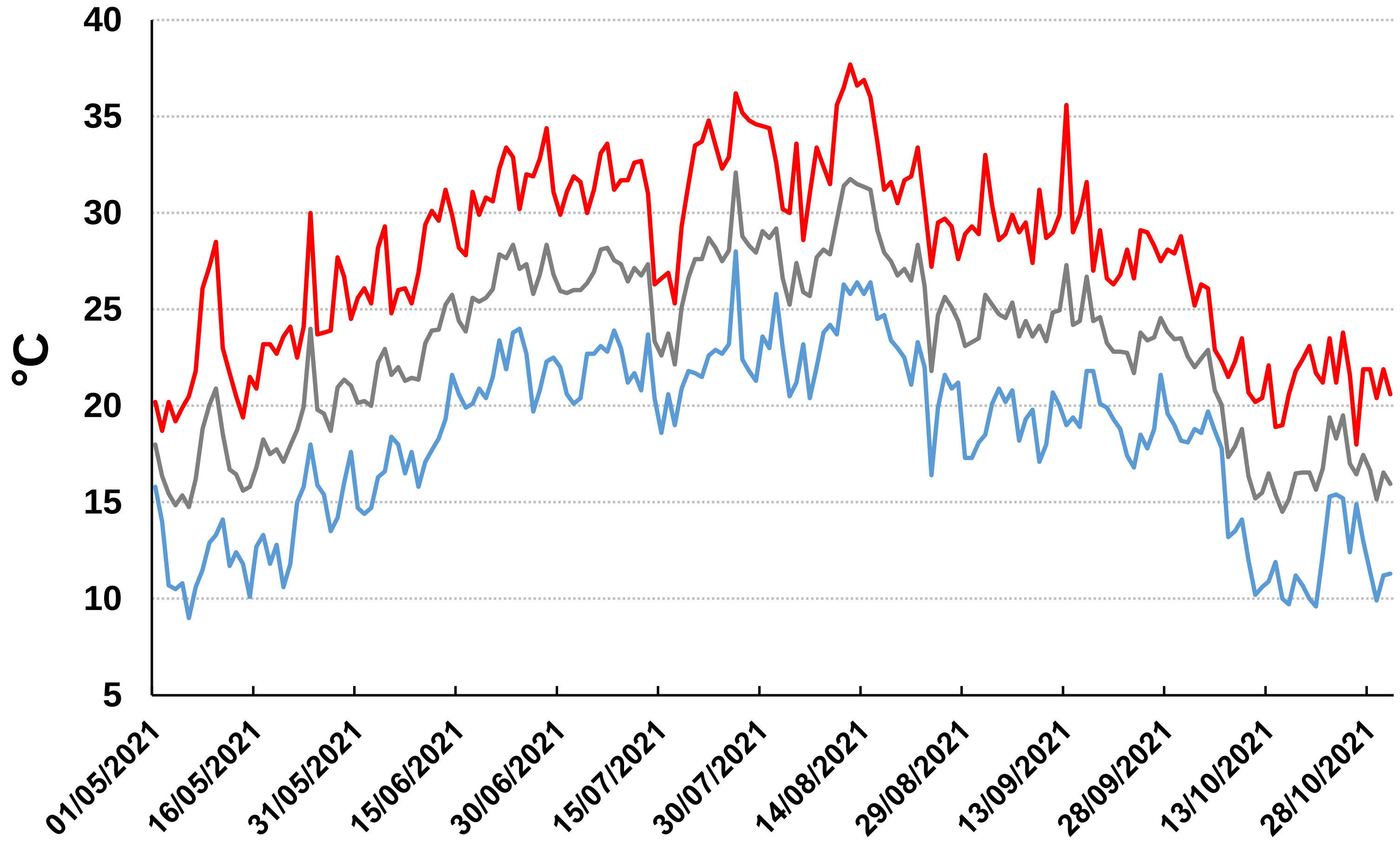

°C
40
35
30
25
20
15
10
5
01/05/2021
16/05/2021
31/05/2021
15/06/2021
30/06/2021
15/07/2021
30/07/2021
14/08/2021
29/08/2021
13/09/2021
28/09/2021
13/10/2021
28/10/2021
