# Supplementary material for: Neofusicoccum mediterraneum Is Involved in a Twig and Branch Dieback of Olive Trees Observed in Salento (Apulia, Italy)
Source: Pathogens. 2022 Jan 2;11(1):53. doi: 10.3390/pathogens11010053 (PMC8780778; doi:10.3390/pathogens11010053)
Supplement: Supplementary file 1 [file pathogens-11-00053-s001.zip › Tex_Table S1.pdf]

Title:

Twig and branch dieback of olive trees in Salento (Apulia, Italy) caused by *Neofusicoccum mediterraneum*

Journal name:

Pathogens - MDPI

Authors:

Angela Brunetti, Antonio Matere, Valentina Lumia, Vittorio Pasciuta, Valeria Fusco, Domenico Sansone, Paolo Marangi, Nicola Cristella, Francesco Faggioli, Marco Scortichini and Massimo Pilotti

Corresponding author:

Massimo Pilotti

Affiliation:

Institution: Council for Agricultural Research and Economics; Department: Research Center for Plant Protection and Certification (CREA-DC) - Via C. G. Bertero 22, 00156 Rome, Italy

E-mail address:

massimo.pilotti@crea.gov.it

Text/Table S1 Methods for DNA extraction, PCR amplifications and sequencing of DNA regions of *Neofusicoccum mediterraneum* isolated from olive trees in Apulia (Italy). In Table A the DNA regions amplified and primers used are reported. In Table B the thermal cycling for each DNA region is described.

Below sequences generated in this study and their accession numbers are also listed.

Genomic DNA (gDNA) of fungal isolates was obtained from 100 mg mycelium sampled from axenic cultures actively growing on sterile cellophane discs (BIO-RAD, Hercules, CA, USA) layered on PDA plates and collected by scraping with a sterile pipette tip. Mycelium samples were powdered with liquid nitrogen, then gDNA was extracted using a DNeasy Plant Mini Kit (Qiagen, Hilden, Germany) following the manufacturer's instructions.

PCR amplifications were carried out on a PCR T100^{TM} Thermal Cycler (BIO-RAD). Reaction assembly was in 50µl volume and contained each primer at 0.2 µM, 2.5 U of High Fidelity Platinum Taq DNA Polymerase (Invitrogen, Thermo Fisher Scientific, Waltham, MA, USA), 0.2 mM (ITS) or 0.4 mM (TUB2 e TEF1-α) of each dNTP, in the buffer supplied by the Taq polymerase manufacturer.

Primer sequences and citations are reported in table A. Thermal cycling is reported in table B.

The PCR products were analysed by agarose gel electrophoresis, excised from gel, and eluted with ISOLATE II PCR and gel kit (Meridian Bioscience, River Hills Drive, Cincinnati, OH, USA) following the manufacturer's instructions. Then amplicons were directly sequenced in both directions by Sanger technology (Bio-Fab research s.r.l. Rome Italy).

Table S1A. DNA regions amplified and primers used

DNA region	Forward primer	Sequence	Reverse primer	Sequence	Citation
ITS	ITS5	5’-GGAAGTAAAAGTCGTAACAAGG-3’	ITS4	5’-TCCTCCGCTTATTGATATGC-3’	[33]
TEF1-α	EF688F	5’-CGGTCACTTGATCTACAAGTG C-3’	EF1251R	5’-CCTCGAACTCACCAGTACCG-3’	[34]
TUB2	T1-β tubulin[1]	5’-AACATGCGTGAGATTGTAAGT-3’	Bt2b[2]	5’-ACCCTCAGTGTAGTGACCCTTGGC-3’	[1] [35]<br>[2] [36]

Table S1B. Thermal cycling (35 cycles) to amplify DNA regions used for fungal identification (Blast and phylogeny)

Loci DNA	Initial denaturation	Denaturation for each cycle	Annealing	Extension	Final extension
ITS	94°C, 3min	94°C, 30s	50°C, 30s	68°C, 1min 15s	68°C, 10min
TEF1-α	94°C, 3min	94°C, 30s	55°C, 45s	68°C, 1min 30s	68°C, 10min
TUB2	94°C, 3min	94°C, 30s	50°C, 30s	68°C, 1min	68°C, 10min

References

33. White, T.J.; Bruns, T.; Lee, S.; Taylor, J. Amplification and direct sequencing of fungal ribosomal RNA genes for phylogenetics. In *PCR protocols: a guide to methods and applications*; Innis, M.A., Gelfand, D.H., Sninsky, J.J., White, T.J., Eds.; Academic Press Inc., London, United Kingdom, 1990; pp. 315-322. Doi: 10.1016/B978-0-12-372180-8.50042-1
34. Alves, A.; Crous, P.W.; Correia, A.; Phillips, A.J.L. Morphological and molecular data reveal cryptic speciation in Lasiodiplodia theobromae. *Fungal Divers.* **2008,** *28,* 1-13.
35. Glass, N.L.; Donaldson, G. Development of primer sets designed for use with PCR to amplify conserved genes from filamentous ascomycetes. *Appl. Environ. Microbiol.* **1995,** *61*, 1323-1330. Doi: 10.1128/aem.61.4.1323-1330.1995
36. O'Donnell, K.; Cigelnik, E. Two divergent intragenomic rDNA ITS2 types within a monophyletic lineage of the fungus fusarium are nonorthologous. *Mol. Phylogenet. Evol.* **1997,** *7(1)*, 103-116. Doi: 10.1006/mpev.1996.0376


```
Sequences:

>OL454501_CREA-DC TPR OL.427_ITS
TTTCCGTAGGTGAACCTGCGGAAGGATCATTACCGAGTTGACTCGAGCTCCGGCTCGACTCTCCCACCCTATGTGTACCT
ACCTCTGTTGCTTTGGCGGGCCGCGGTCCTCCGCACCGGCCCCCCTTCGGGGGGCTGGCCAGCGCCCGCCAGAGGACCAC
AAAACTCCAGTCAGTAAACGTCGCAGTCTGAAAAACAAGTTAATAAACTAAAACTTTCAACAACGGATCTCTTGGTTCTG
GCATCGATGAAGAACGCAGCGAAATGCGATAAGTAATGTGAATTGCAGAATTCAGTGAATCATCGAATCTTTGAACGCAC
ATTGCGCCCCTTGGTATTCCGAGGGGCATGCCTGTTCGAGCGTCATTTCAACCCTCAAGCTCTGCTTGGTATTGGGCTCC
GTCCTCCACGGACGCGCCTCGAAGACCTCGGCGGTGGCGTCTTGCCTCGAGCGTAGTAGAAAACACCTCGCTTTGGAGCG
CACGGCGTCGCCCGCCGGACGAACCTTTGGATTTTTCTCAAGGTTGACCTCGGATCAGGTAGGGATACCCGCTGAACTTA
A

>OL539661_CREA-DC TPR OL.427_TEF1-a
GGTGGTATCGATAAGCGTACCATCGAGAAGTTCGAGAAGGTAAGAAAGCTTTTCCTTCCGCTGCACGCACGCTGGGTGCT
GGGTTGCCGCACTCAATTTGTCTTATCGCTTCGGTGAGGGGCATTTTGGTGGTGGGGTCGGCCCGCGCTAAGCCTTGTTT
GTGCTTCGGCAAAATCTCCACATCTGGTTTTTTGCGACCGGCGTGCGACCGAAGCGCGCCCCTCGCCAGACTCACAGCCG
CACGTGCGACCGGACGCTAACGACCACCAGGAAGCCGCCGAGCTCGGCAAGGGTTCCTTCAAGTACGCCTGGGTTCTTGA
CAAGCTCAAGGCTGAGCGTGAGCGTGGTATCACCATCGACATTGCCCTCTGGAAGTTCGAGACCCCGAAGTACTATGTCA
CCGTCATCGACGCCCCCGGTCACCGTGATTTCATCAAGAACATGATCACTGGTACCTCCCAGGCCGACTGCGCCATTCTC
ATCATTGCCGC

>OL539662_CREA-DC TPR OL.427_TUB2
ACTGCGCCTGCCCGCTTGTTTTCTACGCGTCCCTGACAGCCCCGCGTCTTGCCCCCCGGCTACGACAACGGGGTCAACAC
CGCTGCGCACTCCTGCTAACATCGTCTTTTCGCATCCATAGGTTCACCTCCAGACCGGCCAATGCGTAAGTCTTCTCACA
TCCGCCGAAATCGCTGCGTCGCGCTGACTTTGCCCAGGGTAACCAAATCGGTGCTGCCTTCTGGTTTGTTGCCAAAACAC
TCCCGCTCCCGCGCCCCCGCTGACGCGAATCGACACCACAGGCAGACCATTTCCGGCGAGCACGGCCTGGACGGCTCTGG
CGTGTAAGTCTGCGCCATTTCTTGCGCGAATGGCAATGGCTGACCCGCAGCAGCTACAATGGCACCTCCGACCTCCAGCT
CGAGCGCATGAACGTCTACTTCAACGAGGTACTCTCTCATTAATTGCACAAACACGTAAAGTATGGCAATCTTCTGAACG
CGCAGCAGGCGTCCAACAACAAGTACGTTCCTCGTGCCGTCCTCGTCGACCTCGAGCCCGGCACCATGGATGCCGTCCGC
GCCGGCCCCTTCGGCCAGCTCTTCCGCCCCGACAATTTTGTCTTCGGCCAGTCTGGTGCCGGTAACAACTGG
```
